# A Short History of Trakya University Faculty of Medicine

**DOI:** 10.4274/balkanmedj.galenos.2019.2.0001

**Published:** 2019-02-28

**Authors:** Recep Mesut

Trakya University Faculty of Medicine, located in the west and Turkey’s European territory of Edirne (old Adrianopolis), has been operating in the city. It is 220 km west of İstanbul, 22 km from the Bulgarian border, and 7 km from the Greek border. It was founded by the Roman Emperor Hadrianus in 130 A.D. at the strategic point where the Balkan peninsula meets the three streams (the Meriç, Tunca, and Arda rivers) that give life to the Thracian lands. It was conquered by the Ottomans 92 years before the conquest of İstanbul in 1453 and assumed the starting point and shareholder duties of later European conquests. Its palaces, bridges, mosques, bazaars, fountains, and madrasahs offer extraordinary historical and sacred value for the Turks. The most spectacular Ottoman Mosque (Sultan Selim II Mosque) rises in the middle of the city (Figure 1) (1).

## Sultan II Bayezid Medical Madrasa (1488)

**Figure 2:**
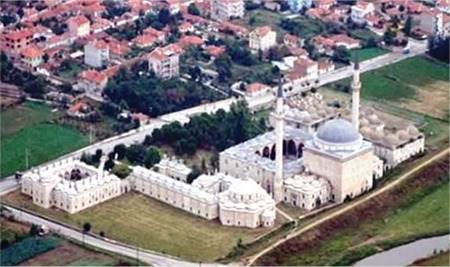
Sultan II Bayezid Complex: Left side Medical Madrasa and Dar-üş-şifa (1488).

In Edirne, which is now an open museum city, very old education and health facilities are still standing, restored and opened to visitors (2). Among these, the Medical madrasa and hospital, which is located in the Sultan II Bayezid Complex, is very famous and notable in Europe (3). Sultan Bayezid II, who was the son of Sultan Mehmed the Conqueror of İstanbul (Constantinopol) and ruled 31 years between 1481 and 1512 (contemporary of Christopher Columbus and Leonardo da Vinci), built a multi-purpose health and social assistance complex on the banks of the Tunca river. Half of the complex was commissioned in 1488 as a hospital (Dar’üş-şifa) and medical school (Medrese-i etıbba) near the mosque (worship), tabhane (hotel), imaret (charity supplying free daily bread) (Figure 2) (4).

Patients were treated free of charge, and the Sultan signed a foundation for the costs of medical boarding students and ordered the transfer of the annual income of the village, shop, mill, field, and garden, which produced a high revenue. The health and social aid complex, which was financially guaranteed, was in service until 1911 (i.e., 423 years) and was the site of physician training. After the transfer of patients to İstanbul in the Balkan War, the First World War, the National Liberation War, and the Second World War made Edirne the border city, and the complex was abandoned and unfunded (5).

**Figure 3:**
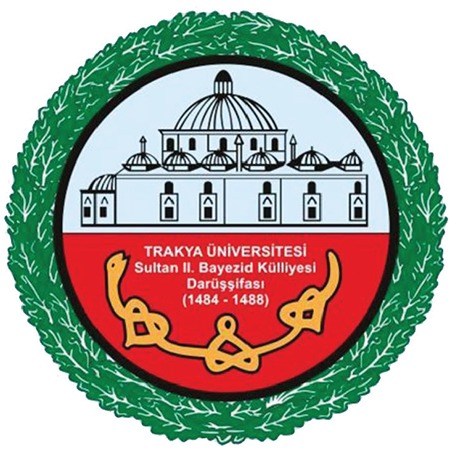
Current emblem of the health museum.

The health-related sections of the complex were transferred to the newly established Trakya University in Edirne in 1984. The university organized “The Museum of Medical History” with an original restoration using its own resources. In 1997, the hospital section, the medrese in 2008 (Medical Faculty) was opened to visitors. The building community with the name of “Trakya University Health Museum” is the only authentic Medical Education Museum at a university in Turkey and the most visited museum in Edirne after Sultan II Selim Mosque. It was also awarded the “2004 Council of Europe Museum Award” for being a bridge between eastern and western cultures. In 2016 it was designated a UNESCO World Heritage Site. Figure 3 shows the current emblem of the health museum (6).

## İstanbul University Edirne Faculty of Medicine (1974)

**Figure 4:**
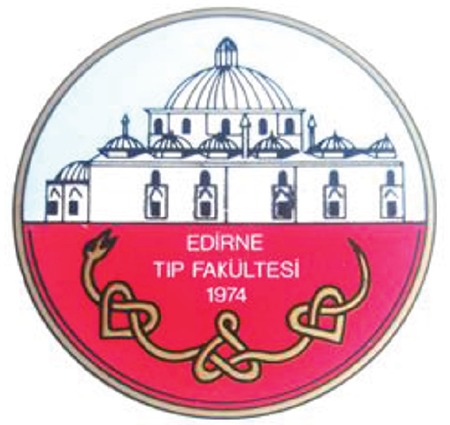
The emblem of Edirne Faculty of Medicine (1974) (Ord. Prof. Dr. A. Süheyl Ünver’s work).

After the war and invasions, when the new Republic of Turkey was formed in 1923, except for madrasah remaining from the Ottoman Empire, there was only one University that offered contemporary university education (İstanbul University). The Republic gradually introduced new higher education institutions and expanded them nationwide. The number of medical faculties offering physician training also increased. İstanbul University founded a second medical faculty (Cerrahpaşa Faculty of Medicine) in 1967. This young faculty also founded the Edirne Faculty of Medicine in 1974 (Figure 4). For eight years (1974-1982) it taught the students of the Edirne Faculty of Medicine and trained the lecturers who will undertake the task in the future—a total of 363 physicians graduated from İstanbul-Cerrahpaşa; 18 faculty members and 36 assistants received their academic formation here (7).

The Faculty of Medicine of Edirne developed as a third medical faculty of administrative and financial management of İstanbul University, with its own Dean and Senator in the Senate, separate budget and staff, and its own staff (13 members), and offices of personnel and student affairs. The Dean’s Office is located in Saraçhane (Horhor Cad. Kavalali Sok. No: 13, Fatih-İstanbul). The 7-person Founders’ Commission, commissioned by the Rector, was formed of voluntary professors and associate professors from Cerrahpaşa Faculty of Medicine. The Founding Dean and high school graduate of Edirne High School, Dr. Suat Vural (1921-2007), was elected and remained in this post for two terms (1975-1978; 1978-1981) (8).

**Figure 5:**
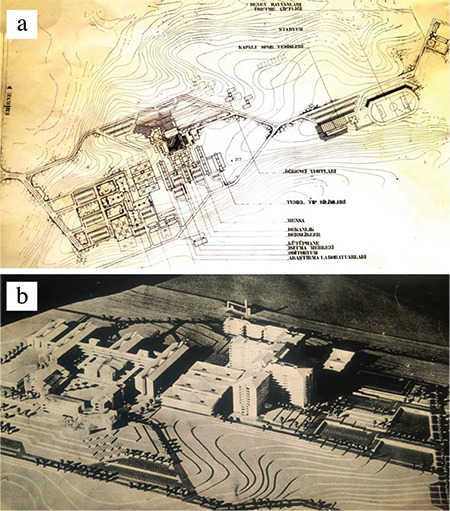
General settlement project (a) and model (b) of the Edirne Faculty of Medicine (1976) (10).

In 1975, the Founding Dean and his accompanying delegation came to Edirne, and the appropriate campus was selected for the Faculty campus “Hadımağa location”, which is 7 km outside the city, in the direction of İstanbul and on the E5 highway. The Sultan II Bayezid Complex, which was built 500 years ago, could not met the requirement of the modern University Hospital and the Faculty of Medicine and there was not enough land in Edirne, which is full of historical monuments. The expropriation activities of the total area of 2,257,290 m^2^ of land and gardens belonging to the municipality and individuals took one year (but most of the people from Edirne who owned the property and the municipality donated their own land) (9). On 8 June 1976, SİSAG Building Designation Group (an institution of Hacettepe Foundations) completed the architectural (Figure 5a, 5b) project of general settlements, infrastructure, and construction, and Prime Minister Süleyman Demirel was inaugurated on 23 August 1976. In the first stage, A-blocks (11 blocks of 6 adjacent blocks, Clinics and Inpatient Services), B-block (3-storey monoblock Polyclinic), and C-block (three-floor emergency service, blood center, and 18 operating rooms) were tendered (a total area of 103,000 m^2^). Due to the lack of appropriation, construction progressed very slowly, and for years under the E5 highway, under rough construction, was exposed to the confused glances of those traveling to Europe (10).

## Trakya University Faculty of Medicine (1982)

In this process, the legal entity of İstanbul University Edirne Faculty of Medicine was disbanded in 1982 and transferred to the newly founded “Trakya University” in Edirne on 20 July 1982. Construction had only reached 25%.

**Figure 6:**
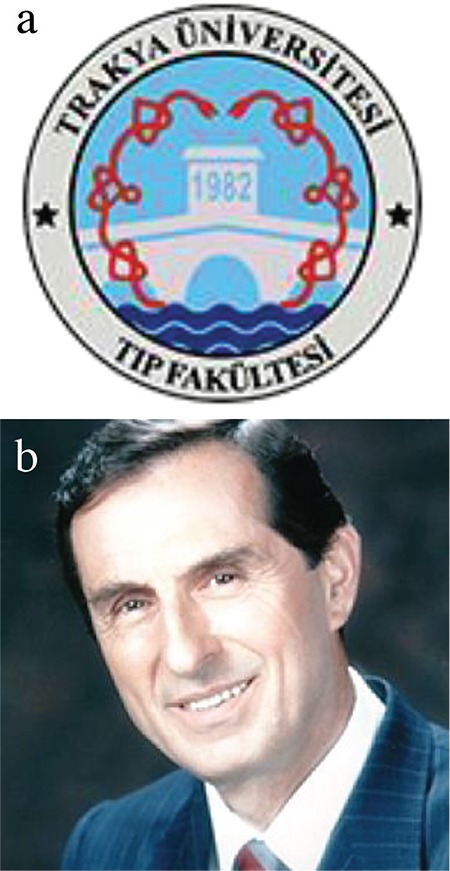
The emblem of Trakya University Faculty of Medicine (1982) (a); The founding rector of Trakya University Dr. Ahmet T. Karadeniz (b).

After the military coup of 12 September 1980, the Higher Education Law (YÖK) of 6 November 1981 was published. Based on this law, on July 20, 1982, a number of new universities were established with Decree Law No: 41. One of them was Trakya University in Edirne. The Edirne Faculty of Medicine was separated from İstanbul University and joined this newly established university, and its name was changed to Trakya University Faculty of Medicine (Figure 6a). Together with the permanent staff, the land in Edirne and the buildings under construction were also transferred. Only the students who were studying in Cerrahpaşa Medical School stayed in İstanbul and graduated in the following years. However, the students who gained entrance to the Edirne Faculty of Medicine in the university entrance exams of 1982 had to go to Edirne. Most of the officers and some of the lecturers did not go to Edirne but resigned or transferred to other offices (7,9).

In 1982, the Trakya University had established, in addition to the Faculty of Medicine, the Faculties of Engineering and Architecture, Science and Letters, and Agriculture in Tekirdağ; two Schools of Higher Education (Edirne, Çanakkale); and four Vocational Schools (Edirne, Tekirdağ, Kırklareli, and Çanakkale). As the Founding Rector (Figure 6b), Dr. Ahmet T. Karadeniz was appointed for 10 years (1982-1992). The founding Dean of the Faculty of Medicine of Trakya was Dr. İsmet Dökmeci (1942, İskilip-2016, Edirne) (8,11).

Prior to the establishment of Trakya University, the following colleges in the city of Edirne provided high school education: the Edirne Education Institute (1969) and the State Academy of Engineering and Architecture (1977). The Edirne Education Institute in the Ayşekadın location, administrative offices and classrooms, a dormitory and dining hall, a gymnasium and a large parcel of land were accelerated to begin the new University. The newly established Faculty of Arts and Sciences and the Faculty of Engineering and Architecture settled here. The Dean of the Faculty of Medicine and Basic Sciences was also present for 1.5 years (11). It was impossible to complete the construction of the Faculty of Medicine outside the city in a short time. All appropriations and workers were directed to the B-block (three-story monoblock polyclinics) building on 10 January 1984. The second semester year of the spring term for Basic Medical Sciences (Anatomy dissection halls, cadaver pools), Histology, Physiology, Biochemistry and Microbiology practice halls, two large classrooms and the Deanship administrative units moved here (9).

**Figure 7:**
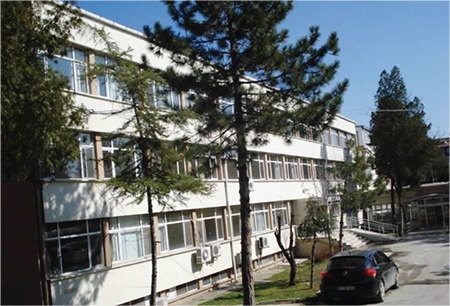
First temporary faculty hospital in the courtyard of the state hospital (1983-1989).

Patient admittance had to start at once. Construction of the additional hospital building (four floors, including the basement) was completed in the courtyard of Edirne State Hospital (Figure 7). The Ministry of Health was requested to allocate this additional hospital building to the Faculty of Medicine. The Ministry of Health responded positively, and the hospital was converted to a temporary faculty hospital with 80 beds. It was opened with a ceremony on 5 June 1983 and served to both patients and students until 1989 (7).

**Figure 8:**
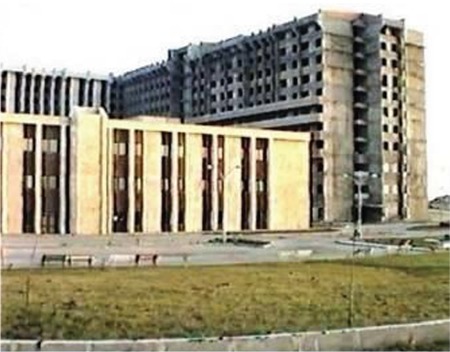
Completed B-block and carcass A-blocks of the Faculty of Medicine (1984).

The construction of the Faculty of Medicine was continued rapidly in the Hadımağa location. The preclinical departments which were going to be taught in the first, second, and third years (Anatomy, Physiology, Histology-Embryology, Biochemistry, Deontology) and Basic Clinical (Pathology, Pharmacology, Microbiology) were placed in B-block temporarily. While the A-blocks and C-blocks were under construction, courses, practices and exams were conducted simultaneously in the B-block (Figure 8).

In 1985, the polyclinics in the city center gradually moved to the campus. In 1986, the laboratories moved. In 1987, the clinic beds were placed in the lower three floors of blocks A1 and A5 (5000 m^2^ closed area), and some of the clinics were placed here. Thus, on the campus, the first stage of the University Hospital was in service with 250 beds.

In 1988, the Amphitheater Block (four amphitheaters with seating for 175 people and four small classrooms with seating for 30 people) was completed in the main project, and polyclinics (B-block) were relieved. In the same year, the construction of the Health Staff School was completed, and a 2-year education college (SHMYO) was put into service. In 1989, the surgical sciences and operating theaters were moved to the campus and the building, which had been used temporarily for 6 years in the city center, was returned to the State Hospital.

In 1990, the second-stage University Hospital (15,000 m^2^ closed area) with 500 beds was in service. Now all the units of the Faculty of Medicine were gathered on the campus outside the city, but the rough construction of the 11-floor A blocks was finished, and detailed workmanship continued.

In 1992, C-block was completed. Eighteen operating rooms with interior fittings and technical equipment were put into service. The entrance floor was allocated to the Emergency Department. The opening ceremony was held on 5 July 1992.

In 1997, the Menza Building, (kitchen and cafeteria) came into service. In this building, spaces for students’ health, social, cultural, and sporting activities were allocated. The D1 and D2 Buildings envisaged for Basic Medical Sciences were completed in 1998, and they moved to the main locations of the Departments in the Polyclinic Building (B-block). Polyclinic units and Radiodiagnostics, Radiotherapy, and Nuclear Medicine were located there. The dialysis unit and cardiovascular surgery were put into service the same year, and open heart surgeries were started.

**Figure 9:**
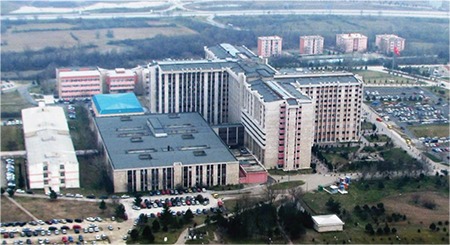
Trakya University Faculty of Medicine (completed in 1999).

In 1999, the construction of all of the 11-floor A-blocks (Inpatient Services, Clinical Departments) was completed (Figure 9). It was opened on 25 November 1999 on the day of salvation of Edirne. The modern University Hospital with 750 beds is in Turkey’s European territory. Twenty-six years ago, Süleyman Demirel, who laid the foundation of the Faculty as the Prime Minister, participated as the President this time and distributed plaques to those who had contributed. In the same year, the indoor sports hall was also put into service.

In 2000, the third block of Basic Medical Sciences (Microbiology, Biochemistry, Biostatistics, and Medical Informatics) was completed. The Central Laboratory moved here. The experimental Animal Center, the Cobalt-60 teletherapy for radiotherapy, and the planning simulator were integrated into the complex in separate buildings.

**Figure 10:**
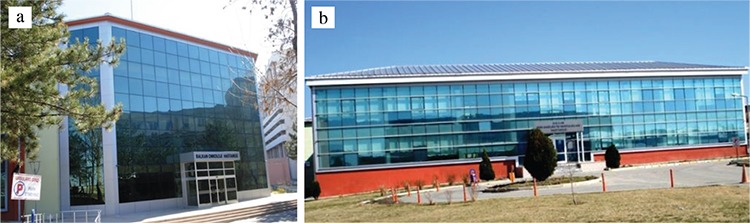
Balkan Oncology (a) and Balkan Psychiatric (b) Hospitals (2012).

In 2004, the central library and the indoor swimming pool were completed and presented to the students and instructors of the campus. In 2009, the campus was called “Balkan Campus 2009”. The rectorate, with all its administrative units, moved to the newly completed modern building at the Faculty of Medicine Campus. The Balkan Convention Center was also opened. In 2010, the Transplantation Center was opened, and in 2011, the first Linak device was established. In 2012, the Oncology Hospital and the Psychiatric Hospital (Balkan Mental Health and Disease Hospital) (AMATEM Center with 20 beds) were moved to separate detached buildings from the main hospital Figure 10.

In 2013, the IVF Center, in 2016 the Cardio-Oncology Unit, in 2017 the Endoscopy Center were established. In 2017, the total capacity of the University Hospitals has reached 1000 beds (130,000 m^2^ closed area). There are 201 faculty members (109 professors, 39 associate professors, and 53 medical professors), 3 lecturers, 8 specialists, and 375 assistants (total 587 members) as academic staff. The annual student quota is over 200. The number of foreign students is 152, of which 6 are medical specialty students. In 2018, Pre-School Medical Education was accredited by the national authority.

As of January 2019, the Faculty of Medicine has the honor of reaching its 5000^th^ graduates and the 40^th^ year of the Balkan Medical Journal (12).

Today, Trakya University Faculty of Medicine, which dates back 530 years, has formed a strong staff of academicians who have been structured in a very short time (36 years) in Edirne, equipped with modern examination and treatment devices. It has become the largest and safest diagnostic, therapeutic and consultative health institution in the Thrace region. Health care services are provided to patients from the Balkan countries as well as physicians and nurses.

## Figures and Tables

**Figure 1:**
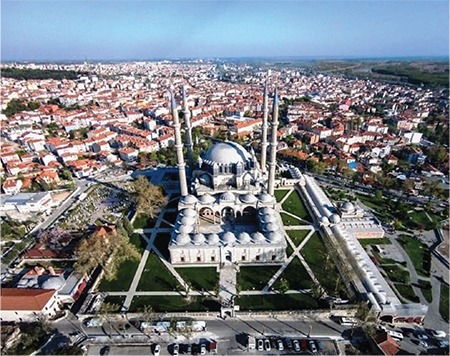
Edirne Selimiye Mosque (1574).
